# Ultra‐thin, high strength, antibiotic‐eluting sutures for prevention of ophthalmic infection

**DOI:** 10.1002/btm2.10204

**Published:** 2020-12-09

**Authors:** Kunal S. Parikh, Revaz Omiadze, Aditya Josyula, Richard Shi, Nicole M. Anders, Ping He, Youseph Yazdi, Peter J. McDonnell, Laura M. Ensign, Justin Hanes

**Affiliations:** ^1^ Center for Nanomedicine The Wilmer Eye Institute, Johns Hopkins University School of Medicine Baltimore Maryland USA; ^2^ Department of Ophthalmology The Wilmer Eye Institute, Johns Hopkins University School of Medicine Baltimore Maryland USA; ^3^ Center for Bioengineering Innovation & Design Johns Hopkins University Baltimore Maryland USA; ^4^ Department of Biomedical Engineering Johns Hopkins University School of Medicine Baltimore Maryland USA; ^5^ Department of Chemical and Biomolecular Engineering Johns Hopkins University Baltimore Maryland USA; ^6^ Department of Oncology Sidney Kimmel Comprehensive Cancer Center, Johns Hopkins University School of Medicine Baltimore Maryland USA

**Keywords:** drug delivery, eye drop, levofloxacin, medical device, nanofiber, ophthalmology, suture

## Abstract

Sutures are applied almost universally at the site of trauma or surgery, making them an ideal platform to modulate the local, postoperative biological response, and improve surgical outcomes. To date, the only globally marketed drug‐eluting sutures are coated with triclosan for antibacterial application in general surgery. Loading drug directly into the suture rather than coating the surface offers the potential to provide drug delivery functionality to microsurgical sutures and achieve sustained drug delivery without increasing suture thickness. However, conventional methods for drug incorporation directly into the suture adversely affect breaking strength. Thus, there are no market offerings for drug‐eluting sutures, drug‐coated, or otherwise, in ophthalmology, where very thin sutures are required. Sutures themselves help facilitate bacterial infection, and antibiotic eye drops are commonly prescribed to prevent infection after ocular surgeries. An antibiotic‐eluting suture may prevent bacterial colonization of sutures and preclude patient compliance issues with eye drops. We report twisting of hundreds of individual drug‐loaded, electrospun nanofibers into a single, ultra‐thin, multifilament suture capable of meeting both size and strength requirements for microsurgical ocular procedures. Nanofiber‐based polycaprolactone sutures demonstrated no loss in strength with loading of 8% levofloxacin, unlike monofilament sutures which lost more than 50% strength. Moreover, nanofiber‐based sutures retained strength with loading of a broad range of drugs, provided antibiotic delivery for 30 days in rat eyes, and prevented ocular infection in a rat model of bacterial keratitis.

## 1 INTRODUCTION

From the first recorded use of natural fibers as sutures in 3500 B.C. to the advent of synthetic, absorbable sutures in 1970, advances in material science and surgical practice have sought to develop the perfect suture: one that is sterile, strong, easy to handle, absorbable, and biologically inert in order to provide the appropriate conditions for wound healing and tissue repair.^1^, ^2^, ^3^, ^4^, ^5^ The ideal suture also degrades and loses strength as the surrounding tissue heals and gains strength, and is able to scale to commercial production. We sought to develop sutures that meet each of these requirements while also providing sufficient and controlled release of a therapeutic moiety in order to improve surgical outcomes.

Sutures are a promising means of therapeutic delivery directly to the surgical site. However, clinical implementation of such technology has been limited due to the inability of drug‐loaded sutures to meet United States Pharmacopeia (U.S.P.) standards for suture strength.^6^, ^7^ Conventional suture manufacturing processes, such as melt extrusion, are not compatible with many therapeutic moieties, and drug‐loaded sutures in preclinical development have demonstrated breaking strength of only 10% of clinical specifications.^4^, ^7^, ^8^, ^9^, ^10^, ^11^, ^12^, ^13^, ^14^, ^15^, ^16^ Drug‐coated sutures have been developed to circumvent these shortcomings, but can be limited in their ability to meet diameter requirements, load sufficient drug, control drug release, and/or scale manufacturing.^17^, ^18^, ^19^, ^20^, ^21^, ^22^, ^23^ Thus, drug‐coated sutures are limited to use in anti‐infection applications, which are the only current market offerings for drug delivery via sutures. However, antibacterial coatings are only available in conjunction with absorbable thread, are only indicated for use in general surgery (U.S.P. sizes 6–0 through 0), and the sutures are too large to meet the respective U.S.P. specifications for diameter.

There is a significant need for an antimicrobial suture in ocular surgery, where, globally, more than 12 million procedures per year use conventional nylon sutures to close ocular wounds and incisions.^24^ Nonabsorbable nylon sutures are a mainstay of ocular surgery due to their biocompatibility and strength retention at the surgical site.^25^ Nylon sutures are used in procedures such as penetrating keratoplasty, where sutures remain in the eye for 12 to 24 months, and as with other implantable devices, increase the risk of infection following ophthalmic procedures due to their susceptibility to bacterial adhesion, proliferation, and biofilm formation.^26^, ^27^, ^28^, ^29^, ^30^, ^31^ Incidence of infectious keratitis following penetrating keratoplasty has been reported between 1.76% and 12.1%.^32^ Suture‐related complications are implicated in 20% to over 50% of these cases, and can have devastating consequences, including poor visual outcomes, reintervention, and graft failure.^28^, ^32^, ^33^, ^34^ Thus, it is particularly important to provide for local antibacterial functionality along with implantation of nonabsorbable sutures within the eye. Local antibiotic delivery from the suture itself would provide bacterial inhibition at the vulnerable surgical incision and help alleviate concerns of noncompliance with topical antibiotic eye drops, which are often prescribed postoperatively. Antibiotic‐eluting sutures may also reduce the need for postoperative oral antibiotic prescriptions, the systemic administration of which can lead to emergence of resistant organisms and associated complications such as *Clostridium difficile* infection and life‐threatening diarrhea.^35^, ^36^ In addition to keratoplasty, glaucoma, retinal detachment, vitrectomy, and cataract surgeries, where nylon sutures have been used, antibiotic‐eluting sutures may also decrease the risk of infection associated with concurrent implantation of keratoprostheses, lacrimal stents, orbital plates, glaucoma drainage implants, or other ocular devices.^37^, ^38^, ^39^, ^40^, ^41^, ^42^, ^43^, ^44^

An antibacterial suture for ophthalmology must be extremely fine (20–50 μm in diameter; U.S.P. sizes 10–0 through 8–0) while retaining high strength for the duration of the intended application and providing sufficient release of an antibiotic agent to reduce or prevent ophthalmic infection.^6^, ^7^ Here, we provide the first demonstration of electrospinning of drug‐loaded nanofibers that are then twisted in a controlled manner to form ultra‐thin, high strength, drug‐eluting sutures of appropriate breaking strength and diameter to meet U.S.P. specifications for ocular surgery.^8^, ^9^, ^13^, ^14^ In order to provide an antibacterial alternative to the use of nylon sutures in ocular surgery, we manufactured sutures composed of polycaprolactone (PCL) and levofloxacin (Levo). Levo is a third‐generation fluoroquinolone and broad‐spectrum ophthalmic antibiotic indicated for treatment of bacterial conjunctivitis.^45^ PCL is a biocompatible polymer capable of long‐term degradation that has been used in sutures and other medical devices for more than 30 years.^25^, ^46^, ^47^, ^48^ We evaluated antibiotic‐eluting suture size, breaking strength, pharmacokinetics, biocompatibility, and efficacy in a rat model of bacterial keratitis.

## 2 RESULTS

We hypothesized that sutures composed of twisted PCL/Levo nanofibers would provide suitable strength at the surgical site for an extended duration while also delivering antibiotic in a sufficient and controlled manner to prevent postoperative suture colonization and ocular infection.

### 2.1 Nanofiber suture manufacture and characterization

**FIGURE 1:**
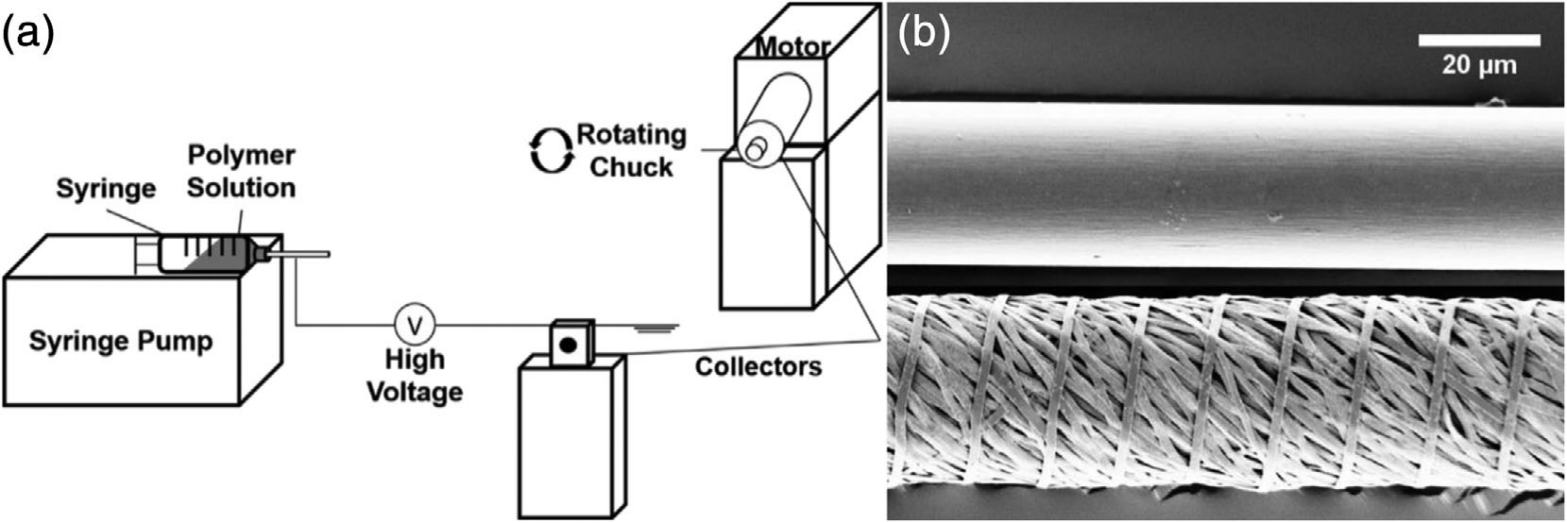
Manufacture of drug‐eluting, multifilament sutures. (a) Schematic of suture manufacturing system. High voltage is applied to a polymer or polymer/drug solution pumped at a controlled rate perpendicular to two grounded collectors. One collector is motorized, allowing for twisting of hundreds of parallel nanofibers into a single, multifilament suture. (b) SEM images of a conventional 10‐0 nylon suture (top) and 10‐0 multifilament PCL/Levo suture (bottom)

In order to limit the effect of drug loading on the strength of polymeric matrices, we engineered a novel manufacturing system capable of producing and twisting together hundreds of individual drug‐loaded, polymeric nanofibers (Figure 1(a)). High voltage was applied to a polymer or polymer/drug solution pumped at a controlled flow rate in order to form polymeric fibers. However, rather than collecting fibers on a rotating drum, which is often employed in electrospinning applications, fibers were collected in parallel between two grounded collectors situated perpendicularly to the syringe pump. Rotation of one collector results in the twisting of deposited parallel fibers into a single 17‐cm‐long multifilament suture. The amount of fiber deposition, and consequently, suture diameter was reproducibly tuned by adjusting spray time.

Seventeen kilovolts were applied to a 10% PCL solution in hexafluoroisopropanol (HFIP) flowing at 450 μL/h for 60 s, followed by twisting of deposited fibers 1575 times in order to manufacture a single, multifilament suture. Scanning electron microscopy (SEM) of multifilament sutures confirmed manufacture of a highly uniform, nonporous, and defect‐free thread composed of nanofibers (Figure 1(b)). Notably, individual nanofibers had a flat, ribbon‐shaped morphology, and had an average width of 729.9 ± 246 nm. Multifilament, drug‐loaded sutures were cylindrical in nature and met U.S.P. specifications for 10‐0 suture diameter (20–29 μm), making them a suitable size for ocular surgery. They were also comparable in both size and shape to commercially available 10‐0 Ethilon® (nylon) sutures (Figure 1(b)).

**FIGURE 2:**
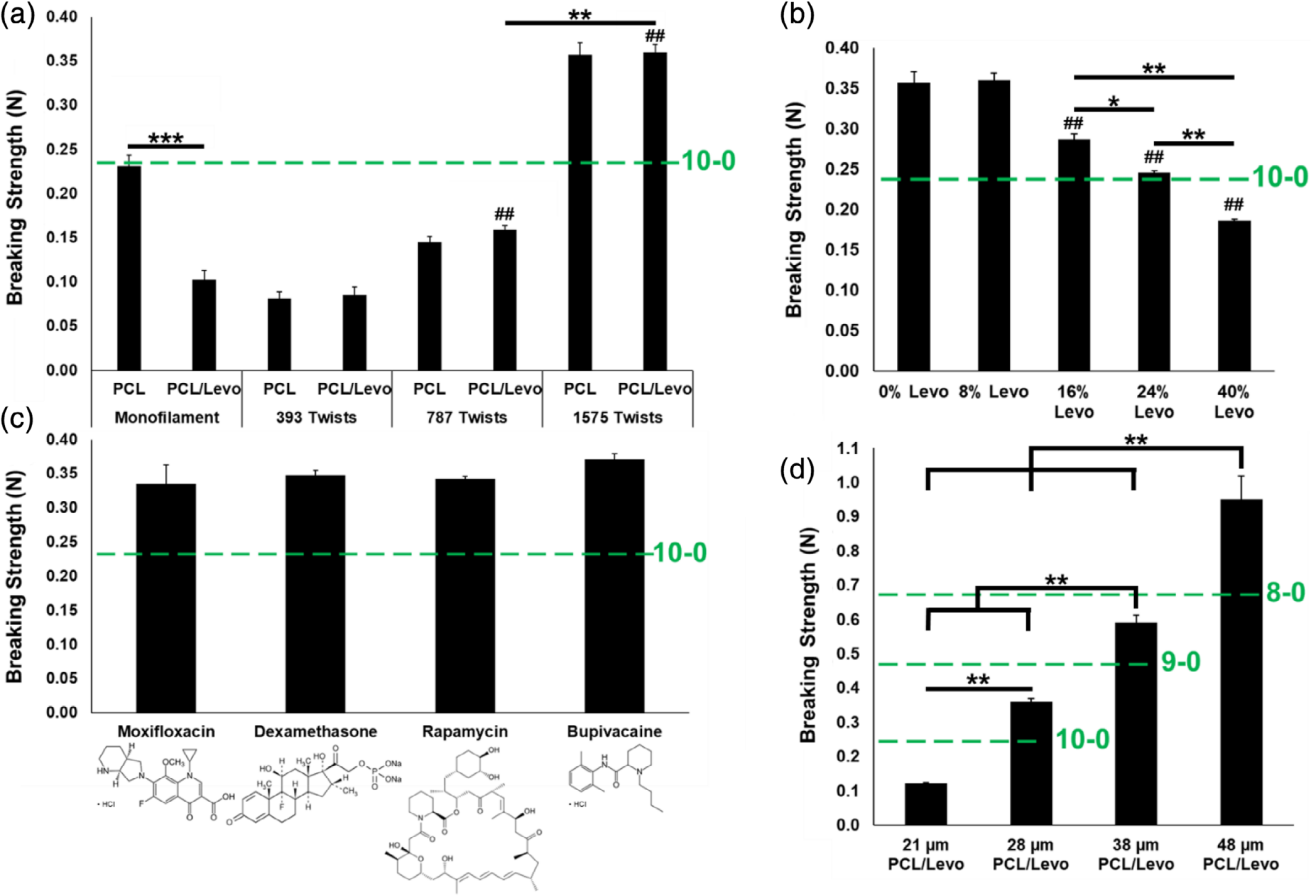
Breaking strength of PCL sutures compared to U.S.P. specifications. (a) Comparison of breaking strength of 28 μm diameter monofilament vs multifilament sutures with varying levels of twisting, with and without 8% Levo, **p < 0.01, ***p < 0.001. ^##^ *p* < 0.01 compared to PCL/Levo monofilament and PCL/Levo 393 twist conditions. (b) Breaking strength of 28 μm PCL/Levo multifilament sutures at 1575 twists with a range of Levo concentrations (0–40%), *p < 0.05, **p < 0.01. ^##^ *p* < 0.01 compared to 0% Levo and 8% Levo conditions. (c) Breaking strength of 28 μm multifilament sutures at 1575 twists composed of PCL and 8% of moxifloxacin hydrochloride, dexamethasone, rapamycin, or bupivacaine hydrochloride. (d) Breaking strength of PCL/8% Levo multifilament sutures with 1575 twists at a range of diameters suitable for ocular surgery (21–48 μm). **p < 0.01. Note that breaking strengths in all figures (a–d) were determined via straight pull. The dashed green line indicates the minimum suture breaking strength for the listed suture gauge size (10‐0 to 8‐0), as defined by the U.S.P. for synthetic absorbable sutures

The principal challenge for translation of drug‐loaded sutures to the clinic has been an inability to meet U.S.P. specifications for suture strength. Thus, we next examined the impact of fiber conformation, drug concentration and type, and suture diameter on suture breaking strength. Figure 2(a) illustrates the difference in PCL suture strength formulated with 8% Levo in either a monofilament or twisted multifilament conformation of identical diameter (28 μm). Monofilament PCL suture breaking strength was reduced by more than 50% (from 0.23 ± 0.01 N to 0.10 ± 0.01 N) when Levo was incorporated (*p* < 0.001). In contrast, multifilament PCL nanofiber suture breaking strength was not significantly changed with drug loading (from 0.36 ± 0.01 to 0.36 ± 0.01 at 1575 twists). Further, multifilament PCL/8% Levo suture breaking strength increased accordingly with the increase in number of collector rotations (from 0.09 ± 0.01 at 393 twists to 0.36 ± 0.01 at 1575 twists). At 1575 twists and 28 μm in diameter, multifilament PCL/8% Levo sutures surpassed the minimum U.S.P. breaking strength specification for synthetic, absorbable, 10‐0‐sized sutures of 0.24 N (Figure 2(a)). Due to the high strength of sutures produced at 1575 twists, all multifilament nanofiber sutures used in later studies were manufactured similarly.

Next, we explored the amount of drug that could be loaded into multifilament nanofiber sutures while maintaining U.S.P. strength specifications. We produced 1575 twist, 28 μm multifilament nanofiber sutures composed of PCL with no drug (0%) or with 8%, 16%, 24%, or 40% Levo within the suture formulation. PCL sutures with 16% or more Levo had a significantly lower breaking strength (*p* < 0.05) than PCL sutures alone or with 8% Levo (Figure 2(b)). It was possible to include up to 24% Levo within the multifilament nanofiber suture formulation while still surpassing clinical strength requirements for a 10‐0 suture. Notably, even with inclusion of 40% Levo into the suture formulation, multifilament PCL nanofiber suture breaking strength was significantly higher (*p* < 0.05) than a monofilament nanofiber suture with 8% Levo (Figure 2(a)), and reached 75% of U.S.P. specification (Figure 2(a)). Eight percent and 16% Levo sutures were selected for use in follow‐on studies as they demonstrated the least amount of suture strength loss with drug loading, while achieving breaking strengths above U.S.P. specifications. Importantly, PCL/8% Levo sutures demonstrated minimal degradation in vitro, and retained 96% of their strength after 31 days and 75% of their strength after 365 days in phosphate buffered saline (PBS) (Table S1), demonstrating potential for long‐term strength retention required for penetrating keratoplasty procedures.

We next evaluated the compatibility of the multifilament nanofiber suture platform with multiple classes of small molecule drugs utilized in ophthalmology and more broadly. We manufactured 1575 twist, 28 μm sutures composed of PCL and 8% of either moxifloxacin hydrochloride, dexamethasone, rapamycin, or bupivacaine hydrochloride. Moxifloxacin HCl (log P = 0.01) is a fourth generation fluoroquinolone with broad antibacterial efficacy currently administered via topical eye drops.^36^, ^49^ Dexamethasone (log P = 1.83) is a corticosteroid used for treatment of uveitis and diabetic macular edema.^50^, ^51^, ^52^, ^53^ Rapamycin (log P = 4.3) is an immunosuppressant and anti‐proliferative agent used in treatment of uveitis.^54^, ^55^, ^56^ Bupivacaine HCl (log P = 3.41) is an analgesic agent that has been used during ocular surgery and for treatment of strabismus.^57^, ^58^, ^59^ Although these prophylactic and therapeutic agents have different molecular weights, ranging from 288.4 to 914.2 g/mol, and physicochemical properties owing to their varying molecular structures, there was no significant difference in breaking strength of multifilament PCL nanofiber sutures loaded with these molecules in comparison to Levo (log P = −0.4) (Figure 2(c)).^60^ Importantly, all drug‐loaded sutures met both size and strength specifications for a 10‐0 suture for ocular surgery.

9‐0 (30–39 μm) and 8‐0 (40–49 μm) sutures are also commonly used in ocular surgery. We evaluated the capacity for our manufacturing platform to scale to larger diameter sutures with correspondingly improved breaking strength by increasing electrospinning spray time to manufacture 1575 twist PCL/8% Levo sutures that were 38 μm (9‐0) and 48 μm (8‐0) in diameter. In order to understand the effect of suture diameter on breaking strength, we also evaluated the breaking strength of 21 μm diameter PCL/8% Levo sutures manufactured in a similar fashion (Figure 2(d)). Varying suture diameter significantly affected breaking strength in all cases (*p* < 0.05). Decreasing suture diameter from 28 to 21 μm decreased breaking strength more significantly than increasing Levo concentration from 8% to 40% (Figure 2(b)), demonstrating the importance of suture diameter in the resulting breaking strength of multifilament nanofiber sutures. Thirty‐eight micrometer PCL/8% Levo multifilament nanofiber sutures (0.59 ± 0.03 N) surpassed U.S.P. specifications for 9‐0 sutures and were 64% stronger than 28 μm sutures (0.36 ± 0.02 N). The breaking strength of 48 μm PCL/8% Levo sutures (0.95 ± 0.07 N), also measured via straight pull, demonstrated a 61% increase in comparison to 38 μm sutures (Figure 2(d)).

### 2.2 In vivo suture biocompatibility

In order to further evaluate the potential use of multifilament nanofiber sutures as an alternative to nylon or other commercially available sutures, we assessed the local tissue reaction to implantation of 3 × 2 mm long 10‐0 nylon, Vicryl®, PCL, PCL/8% Levo, or PCL/16% Levo sutures after 2 days in the rat corneal stroma. There were no gross signs of irritation, inflammation, or infection among any of the treated or control groups (not shown). Histological analysis (Figure S1) revealed that implantation of PCL or PCL/Levo sutures did not cause neovascularization, and that the tissue reaction was comparable to commercially available nylon sutures. Notably, a small ring of immune cells was observed surrounding implanted absorbable Vicryl® sutures (Figure S1).

### 2.3 Pharmacokinetics of levofloxacin delivered from sutures

**FIGURE 3:**
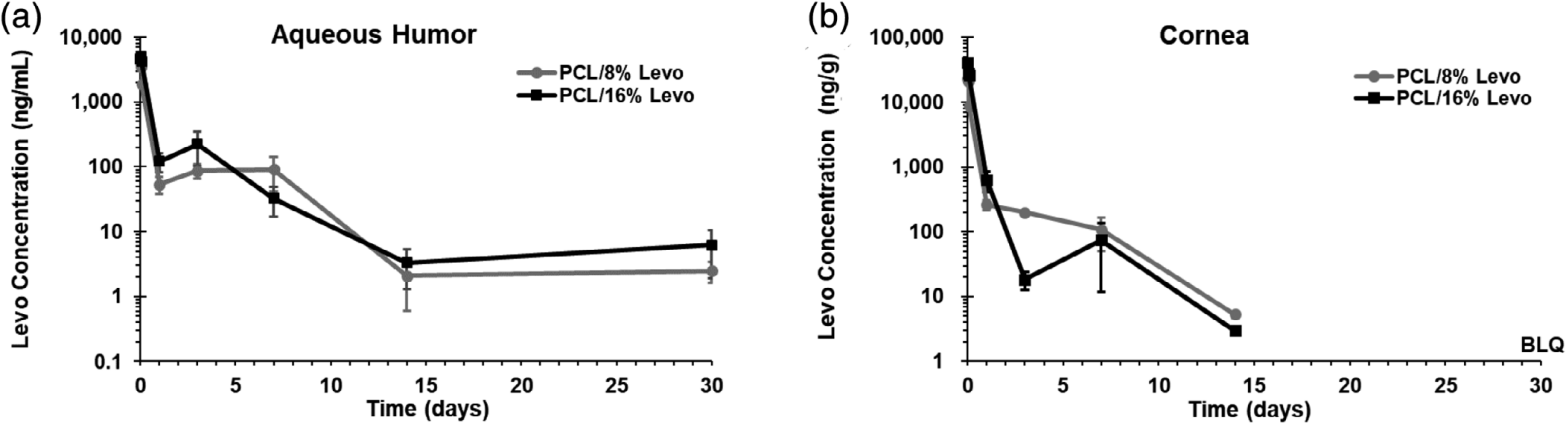
Levofloxacin concentration in rat aqueous humor and corneal tissue over 30 days. Three 2 mm segments of either 10‐0, nanofiber‐based PCL/8% Levo (*n* = 4–8) or PCL/16% Levo (*n* = 4‐8) sutures, respectively, were implanted into rat corneas and removed prior to evaluation of Levo concentration in the aqueous humor and cornea via LC/MS/MS. Eight percent and 16% Levo demonstrate similar concentration profiles throughout the evaluation period. Levo concentrations in the cornea at day 30 were below the limit of quantification (BLQ), indicating values <0.5 ng/g

High‐performance liquid chromatography (HPLC) analysis of dissolved sutures immediately following manufacture revealed Levo loading of 80 and 161 μg/m, respectively, for 8% and 16% Levo sutures. In order to determine the duration and concentration of Levo delivery from sutures in vivo, we conducted a pharmacokinetic study by implanting 3 × 2 mm lengths of 28 μm PCL/8% Levo or PCL/16% Levo sutures into rat corneas. Analysis of Levo concentration in harvested aqueous humor and corneas revealed high levels of antibiotic as quickly as 15 min following suture implantation and for multiple hours afterward (Figure 3). The overall Levo release profiles were similar in eyes implanted with either 8% or 16% Levo sutures. Multifilament nanofiber sutures maintained their location and macroscopic structure throughout the course of the study (not shown), and in both 8% and 16% Levo conditions. Levo was detected in the cornea and aqueous humor 30 days after implantation, although corneal concentrations were below the limit of quantification at day 30, indicating values <0.5 ng/g. Notably, the levels of Levo detected in the aqueous humor was comparable at 14 and 30‐day time points for both suture formulations.

### 2.4 In vivo prevention of ophthalmic infection

**FIGURE 4:**
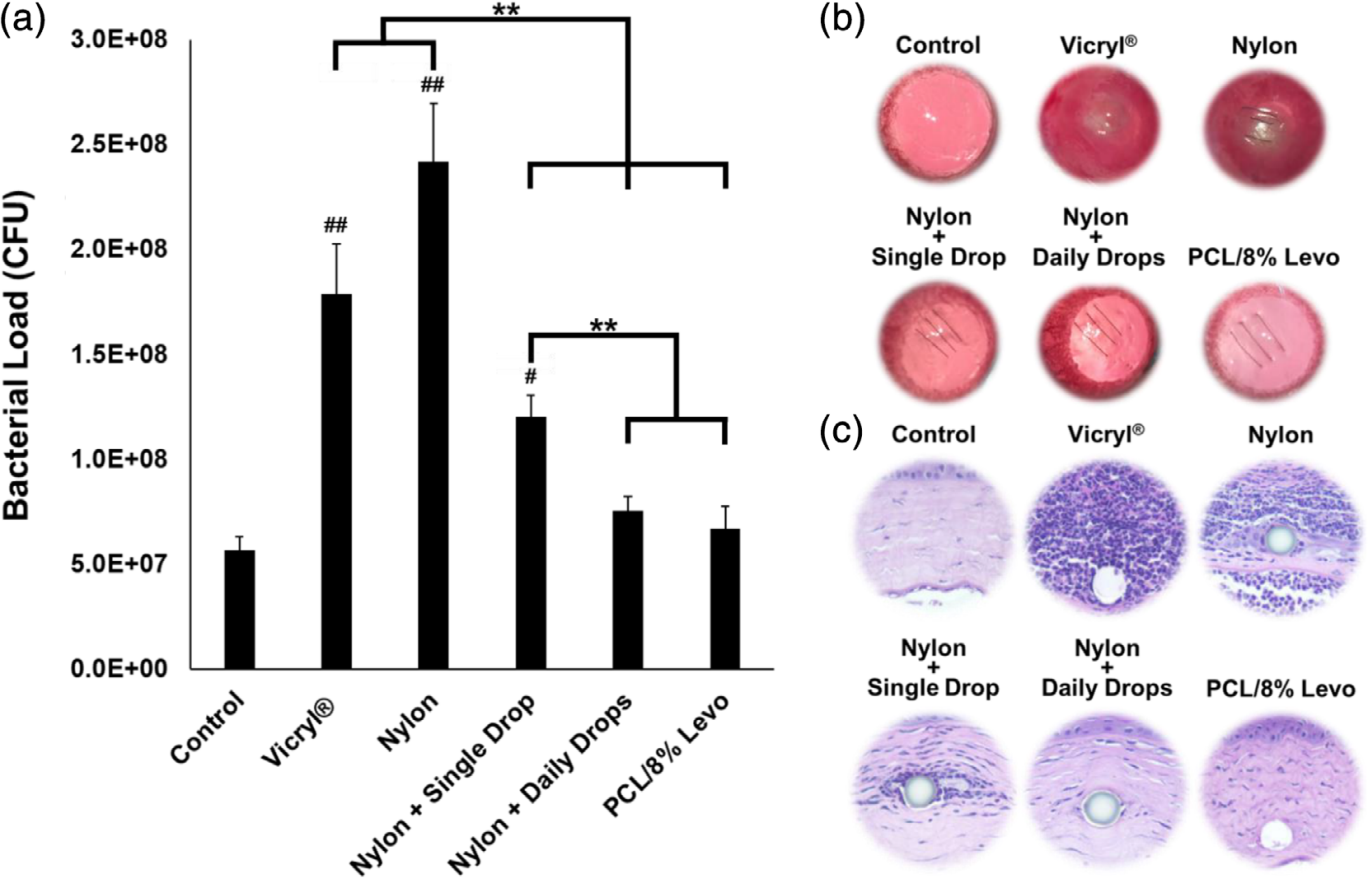
Evaluation of ophthalmic infection 2 days after inoculation of *S. aureus*. (a) Concentration of bacteria in healthy control rat corneas or inoculated corneas containing either Vicryl®, nylon, or nanofiber‐based PCL/8% Levo sutures. Nylon sutures were evaluated alone, with treatment of a single postoperative topical drop of 0.5% Levo, or with treatment of 3 daily drops of 0.5% Levo (*n* = 4, each), **p < 0.01. ^#^ *p* < 0.05, ^##^ *p* < 0.01 compared to control. Representative images of (b) healthy control and experimental eyes 2 days after bacterial inoculation, and (c) H&E stained tissue surrounding sutures 2 days after bacterial inoculation

We next evaluated the capacity of PCL/Levo nanofiber sutures to prevent infection following one or two consecutive inoculations of *Staphylococcus aureus* (*S. aureus*). A bent 30 G needle was used to make three parallel scratches across the surface of the cornea, followed by implantation of three 2 mm lengths of 10‐0 nylon, Vicryl®, or PCL/Levo sutures into the cornea. Subsequently, 100 μL of *S. aureus* was administered to the ocular surface. Rats receiving implantation of nylon sutures were divided into three groups: (1) no postoperative treatment, (2) topical administration of a single drop of 0.5% Levo immediately after inoculation, and (3) topical administration of 0.5% Levo three times daily beginning immediately after inoculation. Implantation of Vicryl® and nylon sutures without postoperative treatment resulted in severe infections characterized by a bacterial load 3.4–4.3 times higher than that of a healthy, control cornea (Figure 4(a)) 2 days after suture implantation and bacterial inoculation. The eyes were highly inflamed and red, with a whitish hue likely indicating bacterial colonization and proliferation surrounding the sutures themselves (Figure 4(b)). Healthy, control corneas contained a small amount of endogenous bacteria, the amount of which was not significantly different than corneas implanted with PCL/8% Levo sutures or corneas implanted with nylon sutures receiving three daily drops of Levo. A single drop of Levo following implantation of nylon sutures significantly decreased the bacterial load in comparison to a nylon suture alone (*p* < 0.05), but was not sufficient to prevent infection (Figure 4(a)). These findings are further confirmed by histological and bacterial culture analysis. Hematoxylin and eosin (H&E) staining revealed substantial inflammation and cellular infiltration within the corneas of rats receiving implantation of Vicryl® or nylon sutures without postoperative administration of Levo (Figure 4(c)). Notably, the density of immune cells was greatest within the immediate vicinity of implanted sutures, providing another indication that the suture itself may be the nidus of infection and location of bacterial adhesion. Immune cells were also concentrated around nylon sutures implanted in rat eyes receiving a single postoperative dose of Levo. However, there was no sign of infection or inflammation in the corneal tissue surrounding PCL/8% Levo nanofiber sutures or nylon sutures in rats receiving three daily eye drop doses of Levo, and the tissue resembled that of a healthy control. Culture of bacterial swabs on agar plates similarly confirmed the presence of infection in rats with implantation of Vicryl® or nylon sutures, or nylon sutures followed by a single dose of Levo administered topically (Figure S2).

**FIGURE 5:**
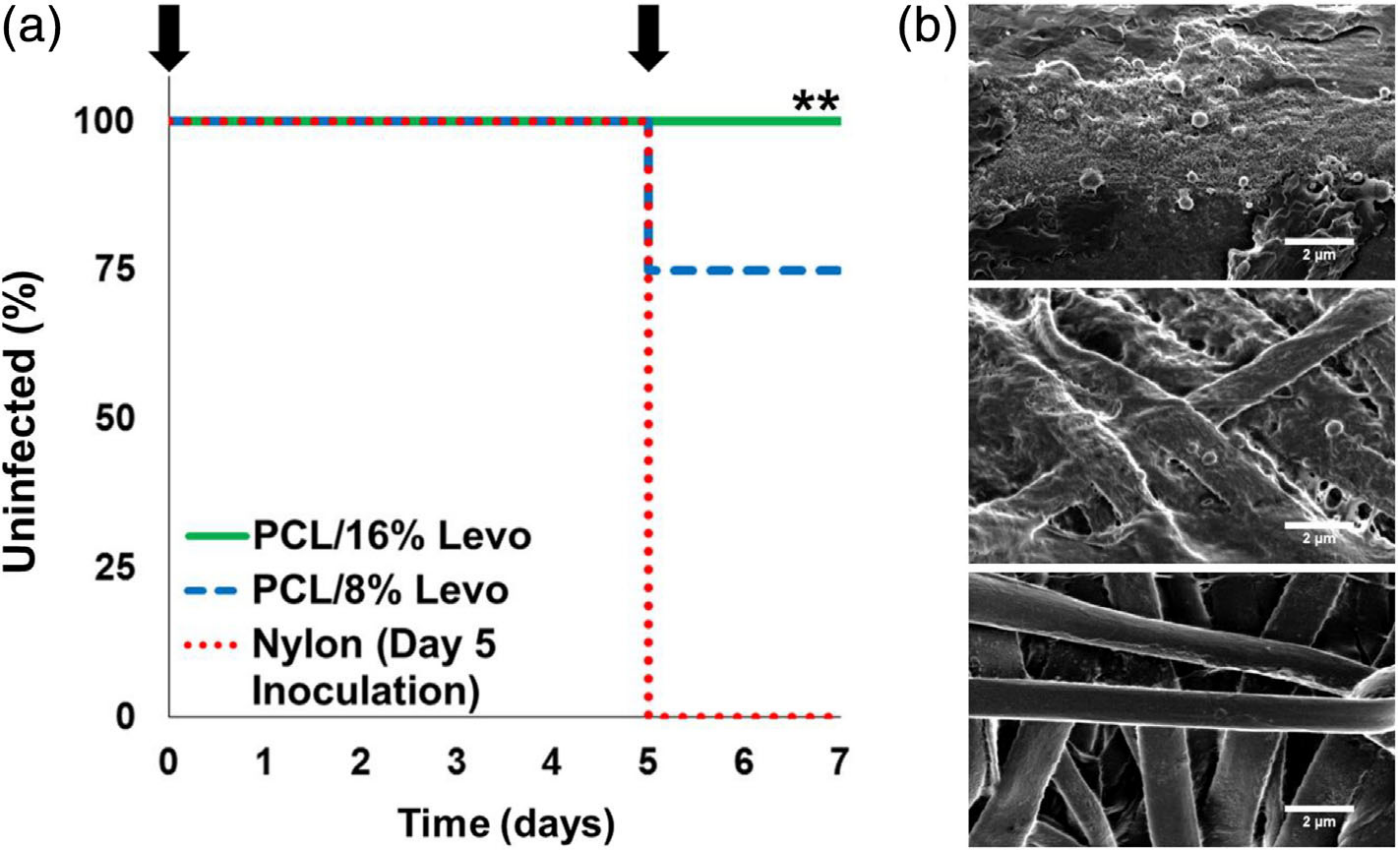
Evaluation of ophthalmic infection following consecutive *S. aureus* inoculations over the period of 1 week. (a) Kaplan–Meier curve indicating prevention of ophthalmic infection following inoculation of *S. aureus* on day 0 and day 5 of Sprague Dawley rat corneas implanted with either nanofiber‐based PCL/8% Levo or PCL/16% Levo sutures (*n* = 8, each). Arrows indicate timing of *S. aureus* inoculation. Note that all sutures were implanted on day 0 and that corneas containing nylon sutures were only inoculated once, on day 5 (*n* = 8). **p < 0.01 compared to nylon (day 5 inoculation), as determined via Mantel–Cox test. (b) High magnification SEM images of nylon (top), PCL/8% Levo (middle), or PCL/16% Levo (bottom) sutures removed from rat corneas on day 7 following one or two consecutive bacterial inoculations

We also evaluated the capacity of 10‐0 PCL/Levo multifilament nanofiber sutures to continue to prevent ocular infection following the immediate postoperative period. PCL/8% Levo and PCL/16% Levo sutures were implanted into rat corneas followed by immediate topical inoculation of *S. aureus* and a second inoculation 5 days after suture implantation. Eyes containing nylon sutures were only inoculated once 5 days after implantation. In congruence with the results of the short‐term infection study, eyes containing PCL/8% Levo or PCL/16% Levo sutures did not become infected after the initial *S. aureus* inoculation. However, 7 days after implantation, 2 of 8 (25%) animals with PCL/8% Levo sutures displayed a minor infection confirmed by bacterial swab and homogenization (Figure S3). Notably, 0 of 8 rat eyes containing 10‐0 PCL/16% Levo sutures showed signs of infection after the second inoculation (Figure 5(a)). These results are significantly different (*p* < 0.01) than the outcomes observed for eyes containing nylon sutures, of which 8 of 8 became infected after a single bacterial inoculation 5 days after suture implantation. SEM images of sutures removed from rat corneas 7 days after implantation further confirmed these results (Figure 5(b)). High magnification images revealed the presence of *S. aureus* on all nylon sutures with biofilm formation. *S. aureus* was also detected on PCL/8% Levo sutures collected from infected eyes, although biofilm formation was less apparent. *S. aureus* colonization was not apparent on PCL/16% Levo sutures collected 7 days after implantation, even after two consecutive bacterial inoculations.

## 3 DISCUSSION

Commercially available sutures are known to be associated with vision‐threatening microbial keratitis and endophthalmitis.^25^, ^26^, ^28^, ^32^, ^33^, ^34^ Suture‐related complications are implicated in up to 50% of infections following penetrating keratoplasty procedures.^28^, ^32^, ^33^, ^34^ Although sutures are employed in a lower percentage of cataract surgeries, after 3 years, more than 65% of sutures have been reported to become loose or broken. Upon removal, cultures reveal that almost 40% of such sutures demonstrate bacterial contamination.^29^ In addition, implantation of devices such as keratoprostheses in conjunction with sutures has demonstrated postoperative infection rates of more than 17%.^61^ An antibiotic‐eluting suture offers an opportunity to reduce or prevent suture‐related postoperative infections, and to reduce the risk of infection following ocular device implantation; however, there are currently no market offerings for drug‐eluting sutures in ophthalmology.

We designed a novel electrospinning system for manufacture of nanostructured, drug‐eluting sutures, and evaluated its potential for development of antibiotic‐eluting sutures for ocular surgery. The platform enables facile fabrication and twisting of aligned, drug loaded nanofibers into ultra‐thin, multifilament sutures of specific diameters. The system is both highly versatile and controlled, allowing for reproducible manufacture of drug‐eluting sutures from a wide range of formulations, and specification of suture diameter via electrospinning spray time and twist number. Notably, the described system enables manufacture of commercial length sutures, which can be lengthened by increasing the distance between the grounded, parallel collectors.

Degradable, multifilament nanofiber sutures fabricated using this manufacturing platform met or exceeded U.S.P. specifications for size and strength suitable for ophthalmic use. The multifilament nanofiber sutures demonstrated biocompatibility comparable to conventional nylon sutures, retained 96% of breaking strength over 31 days, and delivered Levo at detectable levels in rat eyes for at least 30 days. Moreover, Levo‐eluting, multifilament nanofiber sutures manufactured utilizing this platform prevented ocular infection against multiple bacterial challenges for a period of 1 week in vivo, and were significantly more effective than a single postoperative antibiotic drop at decreasing bacterial load within the eye. Further, multifilament nanofiber sutures surpassed breaking strength specifications when loaded with a wide range of small molecule drugs of different physicochemical properties, including antibiotics, steroids, immunosuppressants, and analgesics, indicating broad potential to improve surgical outcomes and alleviate concerns of patient compliance across ophthalmic subspecialty areas and in vascular, plastic, and reconstructive procedures where the postoperative biological response to surgery often leads to complications and reoperations.

To our knowledge, this is the first report of drug‐loaded sutures that surpass U.S.P. breaking strength specifications.^4^, ^8^, ^9^, ^13^ Similar to prior reports in the literature, micron‐sized electrospun PCL monofilament sutures lost more than 50% of their strength upon inclusion of Levo. In contrast, twisted, multifilament nanofiber sutures did not lose strength with inclusion of an equivalent amount of Levo. PCL is a semicrystalline, hydrophobic, and biodegradable polymer.^47^ It has been shown that the process of electrospinning alone can enhance nanofiber molecular orientation and that PCL nanofibers increase in tensile strength with reduced diameter due to molecular confinement.^62^, ^63^ This phenomenon is not observed in PCL fibers produced via the melt flow extrusion process used to manufacture commercially available sutures today.^62^ Further, PCL crystallinity increases along with a decrease in molecular weight.^47^ We sought to maximize fiber crystallinity, and consequently suture strength, by electrospinning nanofibers composed of low molecular weight PCL. Moreover, twisting of individual nanofibers into a multifilament suture provided additional structural reinforcement, resistance to breakage, and knot security.^6^ The flat, ribbon‐shaped morphology of the individual nanofibers suggests that the twisting process led to stretching of nanofibers, which has also been shown to improve polymer chain alignment and tensile strength.^64^ Increased twisting also resulted in a more compact nanofiber bundle, illustrated by the increased spray time necessary to manufacture sutures of an equivalent diameter at a higher number of twists. Thus, increasing the number of twists allowed for incorporation of a greater number of nanofibers into a single suture, thereby amplifying breaking strength and increasing drug loading capacity. Collectively, these factors contributed to manufacture of drug‐loaded, multifilament nanofiber PCL sutures with unprecedented strength.

The highly aligned and hydrophobic nature of PCL nanofibers manufactured through this process likely partitions the drug and polymer.^9^, ^62^ This may explain the almost equivalent strength of multifilament PCL sutures without drug and with inclusion of 8% Levo or other therapeutic and prophylactic agents with disparate molecular structures. This might also lead to the burst release of a fraction of the encapsulated Levo observed following implantation of drug‐loaded sutures into rat eyes. We hypothesize that prior to PCL degradation, the drug delivery profile of small molecules from multifilament PCL sutures will depend primarily on the solubility and distribution of the drug in the nanofibers. As such, we anticipate that more hydrophobic drugs will have a slower overall release rate than Levo, but may also provide for burst release in the immediate postoperative period.

Although a burst release of antibiotic is critical for the prevention of immediate postoperative infection when wounds or surgical incisions are healing and most vulnerable to bacterial infiltration, sutures may be susceptible to bacterial colonization for as long as they remain implanted.^26^, ^27^, ^28^, ^29^, ^32^, ^33^, ^61^, ^65^, ^66^, ^67^ Given the small diameter of 10‐0‐sized sutures utilized in ophthalmic procedures and the difficulty of manufacturing and coating such sutures, it will be challenging for sutures manufactured via conventional methods to provide sufficient drug delivery over this duration.^19^, ^23^ However, local antibiotic delivery from the drug‐loaded sutures reported here may preclude issues of poor patient compliance with topical eye drops, prevent suture‐related infections that lead to treatment failure and reintervention, reduce the need for oral antibiotic use, decrease the risk of infection associated with implantable ocular devices, and serve as an alternative to the more than 12 million nylon sutures used in ocular procedures each year.^24^, ^28^, ^32^, ^36^, ^61^, ^68^ In order to translate this technology platform for patient use in ophthalmology and beyond, we plan to conduct long‐term studies to evaluate suture strength retention and degradation in vivo while also assessing late‐stage wound sealing and tissue healing.

## 4 MATERIALS AND METHODS

### 4.1 Suture manufacture

Polymer solutions were made via dissolution of 80 kDa PCL (Sigma‐Aldrich, St. Louis, Missouri) alone or with drug in HFIP (Sigma‐Aldrich) by shaking overnight at room temperature. PCL concentration was maintained at 10% (w/w) in relation to solvent for multifilament sutures. Levofloxacin (Sigma‐Aldrich), moxifloxacin HCl (LKT Laboratories, St. Paul, Minnesota), dexamethasone (Carbosynth, San Diego, California), rapamycin (LC Labs, Woburn, Massachusetts), and bupivacaine (Carbosynth) were dissolved at either 8%, 16%, 24%, or 40% (w/w) in relation to polymer. Solutions were electrospun via pumping at 450 μL/h through a 20 G blunt‐tip needle with an applied voltage of 17 kV, at a distance of 13 cm from a set of parallel grounded collectors to form 17‐cm‐long parallel nanofibers. One collector was then rotated clockwise 1575 times, unless otherwise specified, prior to removal of the suture from the collectors. Electrospinning time was 30, 60, 90, and 135 seconds for 21, 28, 38, and 48 μm diameter multifilament sutures, respectively. Monofilament sutures were manufactured via application of 5 kV to a 15% PCL solution (w/w) in HFIP flowing at 1 mL/h toward a static, grounded collector 15 cm away.

### 4.2 Suture characterization

#### 4.2.1 Size

Suture diameter was determined via light microscopy using the 20× objective of an Eclipse TS100 (Nikon Instruments, Melville, New York) and calibrated Spot 5.2 Basic imaging software (Spot Imaging, Sterling Heights, Michigan). Each suture was measured at three different locations at least 2 cm apart, and used in additional experimentation only if the average diameter was within ±0.5 μm of the specified diameter.

#### 4.2.2 Morphology

Suture morphology was observed via SEM at 1 kV using a LEO Field Emission SEM (Zeiss, Oberkochen, Germany). Prior to imaging, samples were desiccated and then sputter coated with 10 nm of Au/Pd (Desk II, Denton Vacuum, Moorestown, New Jersey). Measurement of individual nanofiber diameters (*n* = 9) was conducted using ImageJ (US National Institutes of Health, Bethesda, Maryland, https://imagej.nih.gov/ij/).

#### 4.2.3 Breaking strength

Sutures (*n* = 3–4 for each condition) were clamped vertically and then pulled until breaking at a rate of 2.26 mm/min using a DMA 6800 (TA Instruments, Timonium, Maryland). Breaking strength is defined as the load required (in N) to break the suture.

#### 4.2.4 Strength retention

PCL/8% Levo and PCL/16% Levo sutures (*n* = 5) were sectioned into two halves. The breaking strength of one segment was measured as described above, while the other segment was submerged in 1× Dulbecco's Phosphate Buffered Saline (ATCC, Manassas, Virginia) and shaken at 225 rpm at 37°C for 31 days. Sutures were then dried prior to measuring breaking strength.

#### 4.2.5 Drug loading

Levo loading was determined by submerging 15 mm of suture in acetonitrile and sonicating for 30 min prior to evaluation via HPLC (Waters Corporation, Milford, Massachusetts). Samples were injected into a SymmetryTM 300 C18 5 μm column (Waters Corporation) with a mobile phase of 0.1% v/v trifluoroacetic acid (Sigma Aldrich) in water:acetonitrile (75:25 v/v, Fisher Scientific) at a flow rate 1 mL/min. Levo elution was detected at an excitation wavelength of 295 nm and emission wavelength of 496 nm.

### 4.3 Animal studies

All animals were cared for and experiments conducted in accordance with protocols approved by the Animal Care and Use Committee of the Johns Hopkins University, in accordance with the ARVO Statement for the Use of Animals in Ophthalmic and Vision Research, and in accordance with the NIH Guide for the Care and Use of Laboratory Animals.

#### 4.3.1 In vivo biocompatibility

Three 2 mm long 10‐0 nylon, Vicryl® (poly[lactic‐co‐glycolic acid]; PLGA) (Ethicon, Somerville, New Jersey) and PCL/Levo suture filaments were implanted into the corneas of 6–8 week old, male Sprague Dawley rats (*n* = 4 for each suture condition; Harlan Laboratories, Frederick, Maryland). Prior to implantation, rats were intraperitoneally anesthetized with a solution of ketamine:xylazine (75:5 mg/kg, Sigma Aldrich) and a drop of 0.5% proparacaine hydrochloride ophthalmic solution (Bausch & Lomb Inc, Tampa, Florida) was applied to the cornea. Following implantation, the rats were evaluated daily for signs of infection, inflammation, or irritation. Two days after implantation, the rats were euthanized and eyes enucleated, fixed in formalin (Sigma Aldrich) for 24 h, embedded in paraffin, cross sectioned, and stained with H&E for histological evaluation.

#### 4.3.2 Pharmacokinetic study

PCL/8% Levo and PCL/16% Levo sutures were implanted into rat corneas as described above (n = 4 for each formulation at each time point). At 15, 60, and 120 min, and at 1, 3, 7, 14, and 30 days, aqueous humor was collected from each eye, followed by removal of implanted sutures and harvesting of the cornea. Tissue and aqueous humor samples were weighed immediately after harvesting. Corneal tissue samples were homogenized in 100 to 150 μL of PBS prior to extraction. The standard curve and quality control samples were prepared in PBS as a surrogate matrix for both aqueous humor and homogenized tissue. Levo was extracted from 15 μL of aqueous humor or tissue homogenate with 50 μL of acetonitrile containing 1 μg/mL of the internal standard, moxifloxacin‐d4 (Toronto Research Chemicals, Canada). After centrifugation, the supernatant was then transferred into autosampler vials for LC/MS/MS analysis. Separation was achieved with an Agilent Zorbax XDB‐C18 (4.6 × 50 mm, 5 μm) column with water/acetonitrile mobile phase (40:60, v:v) containing 0.1% formic acid using isocratic flow at 0.3 mL/min for 3 min. The column effluent was monitored using an AB Sciex triple quadrupole™ 5500 mass‐spectrometric detector (Sciex, Foster City, California) using electrospray ionization operating in positive mode. The spectrometer was programmed to monitor the following MRM transitions: 362.0 → 318.0 for Levo and 406.1 → 108.0 for the internal standard, moxifloxacin‐d4. Calibration curves for Levo were computed using the area ratio peak of the analysis to the internal standard by using a quadratic equation with a 1/x^2^ weighting function using two different calibration ranges of 0.25 to 500 ng/mL with dilutions up to 1:10 (v:v) and 5 to 5000 ng/mL. Tissue samples volumes (ng/g) were determined via multiplication of the nominal concentration (ng/mL) and dilution factor. The Grubbs' test was utilized to determine and remove outliers at *p* < 0.01.

#### 4.3.3 Bacterial inoculation and evaluation

Sprague Dawley rats were anesthetized as described above. The operative eye was then scratched using a 20 G needle (Fisher Scientific) prior to implantation of three 2 mm long nylon (*n* = 12), Vicryl® (*n* = 4), or PCL/8% Levo (*n* = 4) suture filaments. Nylon sutures are commonly used for corneal transplant and ocular trauma surgeries, while Vicryl sutures are commonly used for cataract procedures. A 100 μL droplet containing 1 × 10^8^ CFU/mL of *S. aureus* was then applied to the ocular surface, left in place for 10 mins, and then removed with a sterile wick without touching the eye. Ten microliter of 0.5% Levo (w/v, the concentration in commercially available eye drops) solution was administered topically either once postoperatively, as would be done by the surgeon, or three times daily, as would be prescribed for prophylaxis, to rat eyes with nylon sutures (*n* = 4, each). Two days after implantation, gross images were taken of each eye, prior to swabbing the cornea with a cotton‐tipped applicator (Fisher Scientific), and streaking onto tryptic soy agar (Fisher Scientific) plates. Plates were stored in an incubator at 37°C for 24 h and then imaged. After swabbing the eye, eyes were enucleated and either prepared for histological evaluation as described above (*n* = 3 for each condition) or evaluated for bacterial load (*n* = 4 for each condition). Briefly, each eye was placed in sterile tryptic soy broth (Fisher Scientific) and homogenized using a Power Gen 125 homogenizer (Fisher Scientific) for 4 min. Samples were then centrifuged at 300 rcf for 5 min, and optical density of the supernatant was measured at a wavelength of 600 nm using a Synergy Mx microplate reader (Biotek, Winooski, Vermont). The bacterial load was determined by subtracting the optical density of fresh tryptic soy broth from experimental values prior to applying a conversion of 0.1 OD to 10^8^ CFU/mL. Infection was confirmed by a positive swab culture and bacterial load significantly higher than a nonoperated control eye.

Alternatively, nylon, PCL/8% Levo, and PCL/16% Levo sutures (*n* = 8, each) were implanted into rat corneas on day 0, with inoculation of *S. aureus* to only the PCL/Levo suture conditions, as described above. On day 2, rat corneas were swabbed to evaluate infection. On day 5, the corneas of all rats were scratched and inoculated with *S. aureus*. On day 7, swabs were taken of each cornea followed by either histological evaluation, bacterial homogenization, or removal of sutures for examination via SEM (*n* = 4 for each condition). For the latter experiment, sutures were removed from the cornea and fixed in formalin (Sigma‐Aldrich) for 30 min prior to washing with PBS and dehydration with increasing concentrations of ethanol (Fisher Scientific). Sutures were then imaged by SEM as described above.

### 4.4 Statistical analysis

Suture breaking strength, Levo concentration, and bacterial load are presented as mean ± standard error. Statistical significance for breaking strength and bacterial load data was determined via one‐way ANOVA followed by Tukey test. Statistical significance for the Kaplan–Meier curve of long‐term infection prevention was determined via the Mantel‐Cox test. Statistical significance is shown as *p* < 0.05 (^#^ or *), *p* < 0.01 (^##^ or **), or ***p < 0.001. Outliers in pharmacokinetics data were determined via Grubbs' test at *p* < 0.01, resulting in removal of three outliers in the D7 data, one each in the aqueous and cornea data in the PCL/Levo 8% group, and one in the cornea data in the PCL/Levo 16% group.

## 5 CONCLUSIONS

Implantation of sutures or other devices into the eye increases the risk of potentially sight‐threatening ophthalmic infection. The surfaces of these devices are vulnerable to bacterial colonization and proliferation, and biofilm formation. We have developed nanostructured, multifilament sutures that can be loaded with high levels of small molecule drugs, and yet, retain high breaking strength, as required by U.S.P. standards. The platform is compatible with several ophthalmic antibiotics of varying physicochemical properties, and surpasses clinical strength requirements while delivering sufficient levels of antibiotic locally. Multifilament nanofiber sutures demonstrated biocompatibility and prevention of ophthalmic infection following multiple inoculations of *S. aureus* over a period of 1 week. This nanostructured, drug‐eluting suture platform holds potential to improve clinical outcomes across a broad spectrum of surgical procedures.

## AUTHOR CONTRIBUTIONS

**Kunal Parikh:** Conceptualization; formal analysis; investigation; methodology; project administration; validation; visualization; writing‐original draft; writing‐review and editing. **Revaz Omiadze:** Conceptualization; investigation; methodology; project administration; validation. **Aditya Josyula:** Conceptualization; formal analysis; investigation; methodology; validation; writing‐review and editing. **Richard Shi:** Investigation; methodology. **Nicole Anders:** Investigation; methodology; validation; writing‐original draft. **Ping He:** Investigation; methodology; validation. **Youseph Yazdi:** Supervision; writing‐review and editing. **Peter McDonnell:** Conceptualization; funding acquisition; supervision; writing‐review and editing. **Laura Ensign:** Conceptualization; funding acquisition; methodology; project administration; supervision; writing‐review and editing. **Justin Hanes:** Conceptualization; funding acquisition; project administration; supervision; writing‐review and editing.

## CONFLICT OF INTEREST

Kunal S. Parikh, Laura M. Ensign, and Justin Hanes have filed patent applications regarding the subject matter of this article. The authors have no other conflicts of interest to declare.

## Supporting information


**Appendix** S1: Supporting information

## Data Availability

The data that support the findings of this study are available from the corresponding authors upon reasonable request.

## References

[1] Snyder CC . On the history of the suture. Bull Hist Dent. 1977;25(2):79‐84. 346101

[2] Spotnitz WD , Falstrom JK , Rodeheaver GT . The role of sutures and fibrin sealant in wound healing. Surg Clin North Am. 1997;77(3):651‐669. 9194885 10.1016/s0039-6109(05)70573-9

[3] Neligan PC . Bioactive sutures. Plast Reconstr Surg. 2006;118:1645‐1647. 17102740 10.1097/01.prs.0000248418.23513.1f

[4] Hu W , Huang ZM , Liu XY . Development of braided drug‐loaded nanofiber sutures. Nanotechnology. 2010;21(31):315104. 20622298 10.1088/0957-4484/21/31/315104

[5] Pillai CK , Sharma CP . Review paper: absorbable polymeric surgical sutures: chemistry, production, properties, biodegradability, and performance. J Biomater Appl. 2010;25(4):291‐366. 20971780 10.1177/0885328210384890

[6] Pruitt LA , Chakravartula AM . Mechanics of biomaterials: fundamental principles for implant design. MRS Bull. 2012;37(7):698.

[7] Kashiwabuchi F , Parikh KS , Omiadze R , et al. Development of absorbable, antibiotic‐eluting sutures for ophthalmic surgery. Transl Vis Sci Technol. 2017;6(1):1‐1. 10.1167/tvst.6.1.1 PMC5225995 28083445

[8] Padmakumar S , Joseph J , Neppalli MH , et al. Electrospun polymeric core–sheath yarns as drug eluting surgical sutures. ACS Appl Mater Interfaces. 2016;8(11):6925‐6934. 26936629 10.1021/acsami.6b00874

[9] Weldon CB , Tsui JH , Shankarappa SA , et al. Electrospun drug‐eluting sutures for local anesthesia. J Control Release. 2012;161(3):903‐909. 22609349 10.1016/j.jconrel.2012.05.021 PMC3412890

[10] Serrano C , Garcia‐Fernandez L , Fernandez‐Blazquez JP , et al. Nanostructured medical sutures with antibacterial properties. Biomaterials. 2015;52:291‐300. 25818435 10.1016/j.biomaterials.2015.02.039

[11] Chang HI , Lau YC , Yan C , Coombes AG . Controlled release of an antibiotic, gentamicin sulphate, from gravity spun polycaprolactone fibers. J Biomed Mater Res A. 2008;84(1):230‐237. 17607742 10.1002/jbm.a.31476

[12] Valarezo E , Stanzione M , Tammaro L , Cartuche L , Malagon O , Vittoria V . Preparation, characterization and antibacterial activity of poly(epsilon‐caprolactone) electrospun fibers loaded with amoxicillin for controlled release in biomedical applications. J Nanosci Nanotechnol. 2013;13(3):1717‐1726. 23755579 10.1166/jnn.2013.7119

[13] He C‐L , Huang Z‐M , Han X‐J . Fabrication of drug‐loaded electrospun aligned fibrous threads for suture applications. J Biomed Mater Res A. 2008;89A:80‐95. 10.1002/jbm.a.32004 18428982

[14] Hu W , Huang Z‐M , Liu X‐Y . Development of braided drug‐nanofiber sutures. Nanotechnology. 2010;21:1‐11. 10.1088/0957-4484/21/31/315104 20622298

[15] Champeau M , Thomassin J‐M , Tassaing T , Jerome C . Drug loading of sutures by supercritical CO_2_ impregnation: effect of polymer/drug interactions and thermal transitions. Macromol Mater Eng. 2015;300(6):596‐610.

[16] Catanzanoa O , Aciernob S , Russo P , et al. Melt‐spun bioactive sutures containing nanohybrids for local delivery of anti‐inflammatory drugs. Mater Sci Eng C. 2014;43:300‐309. 10.1016/j.msec.2014.07.012 25175217

[17] Lee D‐H , Kwon T‐Y , Kim K‐H , et al. Anti‐inflammatory drug releasing absorbable surgical sutures using poly(lactic‐co‐glycolic acid) particle carriers. Polym Bull. 2014;71(8):1933‐1946.

[18] Janiga P , Elayarajah B , Rajendran R , Rammohan R , Venkatrajah B , Asa S . Drug‐eluting silk sutures to retard post‐operative surgical site infections. J Ind Text. 2012;42(2):176‐190.

[19] Obermeier A , Schneider J , Wehner S , et al. Novel high efficient coatings for anti‐microbial surgical sutures using chlorhexidine in fatty acid slow‐release carrier systems. PLoS One. 2014;9(7):e101426. 24983633 10.1371/journal.pone.0101426 PMC4077814

[20] Ming X , Rothenburger S , Nichols MM . In vivo and in vitro antibacterial efficacy of PDS plus (polidioxanone with triclosan) suture. Surg Infect (Larchmt). 2008;9(4):451‐457. 18687027 10.1089/sur.2007.061

[21] Bigalke C , Luderer F , Wulf K , et al. VEGF‐releasing suture material for enhancement of vascularization: development, in vitro and in vivo study. Acta Biomater. 2014;10(12):5081‐5089. 25204522 10.1016/j.actbio.2014.09.002

[22] Ming X , Rothenburger S , Yang D . In vitro antibacterial efficacy of MONOCRYL plus antibacterial suture (Poliglecaprone 25 with triclosan). Surg Infect (Larchmt). 2007;8(2):201‐208. 17437365 10.1089/sur.2006.005

[23] Lee JE , Park S , Park M , et al. Surgical suture assembled with polymeric drug‐delivery sheet for sustained, local pain relief. Acta Biomater. 2013;9(9):8318‐8327. 23770220 10.1016/j.actbio.2013.06.003

[24] Grinstaff MW . Designing hydrogel adhesives for corneal wound repair. Biomaterials. 2007;28(35):5205‐5214. 17889330 10.1016/j.biomaterials.2007.08.041 PMC3878817

[25] Ulery BD , Nair LS , Laurencin CT . Biomedical applications of biodegradable polymers. J Polym Sci B. 2011;49(12):832‐864. 10.1002/polb.22259 PMC3136871 21769165

[26] Lee BJ , Smith SD , Jeng BH . Suture‐related corneal infections after clear corneal cataract surgery. J Cataract Refract Surg. 2009;35:939‐942. 19393897 10.1016/j.jcrs.2008.10.061

[27] Edmiston CE , Seabrook GR , Goheen MP , et al. Bacterial adherence to surgical sutures: can antibacterial‐coated sutures reduce the risk of microbial contamination. J Am Col Surg. 2006;203:481‐489. 10.1016/j.jamcollsurg.2006.06.026 17000391

[28] Hood CT , Lee BJ , Jeng BH . Incidence, occurrence rate, and characteristics of suture‐related corneal infections after penetrating keratoplasty. Cornea. 2011;30:624‐628. 21282987 10.1097/ICO.0b013e3182041755

[29] Heaven CJ , Davison CR , Cockcroft PM . Bacterial contamination of nylon corneal sutures. Eye (Lond). 1995;9(Pt 1):116‐118. 7713238 10.1038/eye.1995.18

[30] Katz S , Izhar M , Mirelman D . Bacterial adherence to surgical sutures. A possible factor in suture induced infection. Ann Surg. 1981;194(1):35‐41. 7018429 10.1097/00000658-198107000-00007 PMC1345192

[31] Masini BD , Stinner DJ , Waterman SM , Wenke JC . Bacterial adherence to suture materials. J Surg Educ. 2011;68(2):101‐104. 21338964 10.1016/j.jsurg.2010.09.015

[32] Lin IH , Chang Y‐S , Tseng S‐H , Huang Y‐H . A comparative, retrospective, observational study of the clinical and microbiological profiles of post‐penetrating keratoplasty keratitis. Sci Rep. 2016;6:32751. 27587283 10.1038/srep32751 PMC5009354

[33] Moorthy S , Graue E , Jhanji V , Constantinou M , Vajpayee RB . Microbial keratitis after penetrating keratoplasty: impact of sutures. Am J Ophthalmol. 2011;152(2):189‐194.e182. 21624557 10.1016/j.ajo.2011.01.038

[34] Christo CG , van Rooij J , Geerards AJM , Remeijer L , Beekhuis WH . Suture‐related complications following keratoplasty: a 5‐year retrospective study. Cornea. 2001;20(8):816‐819. 11685058 10.1097/00003226-200111000-00008

[35] Centers for Disease Control and Prevention . Antibiotic Resistance Threats in the United States. 2013.

[36] Bremond‐Gignac D , Chiambaretta F , Milazzo SA . European perspective on topical ophthalmic antibiotics: current and evolving options. Ophthalmol Eye Dis. 2011;3:29‐43. 23861622 10.4137/OED.S4866 PMC3661455

[37] Srikumaran D , Munoz B , Aldave AJ , et al. Long‐term outcomes of boston type 1 keratoprosthesis implantation: a retrospective multicenter cohort. Ophthalmology. 2014;121(11):2159‐2164. 25017414 10.1016/j.ophtha.2014.05.030

[38] Kim MJ , Yu F , Aldave AJ . Microbial keratitis after boston type I keratoprosthesis implantation: incidence, organisms, risk factors, and outcomes. Ophthalmology. 2013;120(11):2209‐2216. 23747162 10.1016/j.ophtha.2013.05.001

[39] Ahmad S , Mathews PM , Lindsley K , et al. Boston type 1 keratoprosthesis versus repeat donor keratoplasty for corneal graft failure: a systematic review and meta‐analysis. Ophthalmology. 2016;123(1):165‐177. 26545318 10.1016/j.ophtha.2015.09.028

[40] Samimi DB , Ediriwickrema LS , Bielory BP , Miller D , Lee W , Johnson TE . Microbiology and biofilm trends of silicone lacrimal implants: comparing infected versus routinely removed stents. Ophthal Plast Reconstr Surg. 2016;32(6):452‐457. 10.1097/IOP.0000000000000590 26588208

[41] Samimi DB , Bielory BP , Miller D , Johnson TE . Microbiologic trends and biofilm growth on explanted periorbital biomaterials: a 30‐year review. Ophthal Plast Reconstr Surg. 2013;29(5):376‐381. 10.1097/IOP.0b013e31829a7313 23880975

[42] Nguyen QH , Budenz DL , Parrish Ii RK . Complications of Baerveldt glaucoma drainage implants. Arch Ophthalmol. 1998;116(5):571‐575. 9596491 10.1001/archopht.116.5.571

[43] Al‐Torbak AA , Al‐Shahwan S , Al‐Jadaan I , Al‐Hommadi A , Edward DP . Endophthalmitis associated with the Ahmed glaucoma valve implant. Br J Ophthalmol. 2005;89(4):454‐458. 15774923 10.1136/bjo.2004.049015 PMC1772581

[44] Gedde SJ , Scott IU , Tabandeh H , et al. Late endophthalmitis associated with glaucoma drainage implants. Ophthalmology. 2001;108(7):1323‐1327. 11425695 10.1016/s0161-6420(01)00598-x

[45] Healy DP , Holland EJ , Nordlund ML , et al. Concentrations of levofloxacin, ofloxacin, and ciprofloxacin in human corneal stromal tissue and aqueous humor after topical administration. Cornea. 2004;23(3):255‐263. 15084858 10.1097/00003226-200404000-00007

[46] Dash TK , Konkimalla VB . Poly‐є‐caprolactone based formulations for drug delivery and tissue engineering: a review. J Control Release. 2012;158(1):15‐33. 10.1016/j.jconrel.2011.09.064 21963774

[47] Woodruff MA , Hutmacher DW . The return of a forgotten polymer—polycaprolactone in the 21st century. Prog Polym Sci. 2010;35(10):1217‐1256.

[48] Dash TK , Konkimalla VB . Polymeric modification and its implication in drug delivery: poly‐ε‐caprolactone (PCL) as a model polymer. Mol Pharm. 2012;9(9):2365‐2379. 22823097 10.1021/mp3001952

[49] Stigliani M , Haghi M , Russo P , Young PM , Traini D . Antibiotic transport across bronchial epithelial cells: effects of molecular weight, LogP and apparent permeability. Eur J Pharm Sci. 2016;83:45‐51. 26690046 10.1016/j.ejps.2015.12.010

[50] Laurell CG , Zetterström C . Effects of dexamethasone, diclofenac, or placebo on the inflammatory response after cataract surgery. Br J Ophthalmol. 2002;86(12):1380‐1384. 12446370 10.1136/bjo.86.12.1380 PMC1771439

[51] Haller JA , Bandello F , Belfort R, Jr. , et al. Randomized, sham‐controlled trial of dexamethasone intravitreal implant in patients with macular edema due to retinal vein occlusion. Ophthalmology. 2010;117(6):1134‐1146.e1133. 20417567 10.1016/j.ophtha.2010.03.032

[52] Lowder C , Belfort R Jr , Lightman S , et al. Dexamethasone intravitreal implant for noninfectious intermediate or posterior uveitis. Arch Ophthalmol. 2011;129(5):545‐553. 21220619 10.1001/archophthalmol.2010.339

[53] Paturi J , Kim HD , Chakraborty B , Friden PM , Banga AK . Transdermal and intradermal iontophoretic delivery of dexamethasone sodium phosphate: quantification of the drug localized in skin. J Drug Target. 2010;18(2):134‐140. 19772394 10.3109/10611860903278015

[54] Shanmuganathan VA , Casely EM , Raj D , et al. The efficacy of sirolimus in the treatment of patients with refractory uveitis. Br J Ophthalmol. 2005;89(6):666‐669. 15923497 10.1136/bjo.2004.048199 PMC1772655

[55] Bertelmann E , Pleyer U . Immunomodulatory therapy in ophthalmology – is there a place for topical application? Ophthalmologica. 2004;218(6):359‐367. 15564752 10.1159/000080937

[56] Emoto C , Fukuda T , Cox S , Christians U , Vinks AA . Development of a physiologically‐based pharmacokinetic model for sirolimus: predicting bioavailability based on intestinal CYP3A content. CPT Pharmacometrics Syst Pharmacol. 2013;2(7):e59‐e59. 23884207 10.1038/psp.2013.33 PMC3731827

[57] Borazan M , Karalezli A , Oto S , Algan C , Aydin Akova Y . Comparison of a bupivacaine 0.5% and lidocaine 2% mixture with levobupivacaine 0.75% and ropivacaine 1% in peribulbar anaesthesia for cataract surgery with phacoemulsification. Acta Ophthalmol Scand. 2007;85(8):844‐847. 17662095 10.1111/j.1600-0420.2007.00976.x

[58] Miller JM , Scott AB , Danh KK , Strasser D , Sane M . Bupivacaine injection remodels extraocular muscles and corrects comitant strabismus. Ophthalmology. 2013;120(12):2733‐2740. 23916485 10.1016/j.ophtha.2013.06.003

[59] Sudoh Y , Cahoon EE , Gerner P , Wang GK . Tricyclic antidepressants as long‐acting local anesthetics. Pain. 2003;103(1):49‐55. 12749958 10.1016/s0304-3959(02)00375-5

[60] Frick A , Möller H , Wirbitzki E . Biopharmaceutical characterization of oral immediate release drug products. In vitro/in vivo comparison of phenoxymethylpenicillin potassium, glimepiride and levofloxacin. Eur J Pharm Biopharm. 1998;46(3):305‐311. 9885303 10.1016/s0939-6411(98)00041-1

[61] Wagoner MD , Welder JD , Goins KM , Greiner MA . Microbial keratitis and endophthalmitis after the boston type 1 keratoprosthesis. Cornea. 2016;35(4):486‐493. 26764885 10.1097/ICO.0000000000000738

[62] Wong S‐C , Baji A , Leng S . Effect of fiber diameter on tensile properties of electrospun poly(ɛ‐caprolactone). Polymer. 2008;49(21):4713‐4722.

[63] Ero‐Phillips O , Jenkins M , Stamboulis A . Tailoring crystallinity of electrospun PLLA fibres by control of electrospinning parameters. Polymers. 2012;4(3):1331‐1348.

[64] Zhang S , Liu X , Barreto‐Ortiz SF , et al. Creating polymer hydrogel microfibres with internal alignment via electrical and mechanical stretching. Biomaterials. 2014;35(10):3243‐3251. 24439410 10.1016/j.biomaterials.2013.12.081 PMC3923323

[65] Chen WL , Wu CY , Hu FR , Wang IJ . Therapeutic penetrating keratoplasty for microbial keratitis in Taiwan from 1987 to 2001. Am J Ophthalmol. 2004;137(4):736‐743. 15059714 10.1016/j.ajo.2003.11.010

[66] Callegan MC , Engelbert M , Parke DW 2nd , Jett BD , Gilmore MS . Bacterial endophthalmitis: epidemiology, therapeutics, and bacterium‐host interactions. Clin Microbiol Rev. 2002;15(1):111‐124. 11781270 10.1128/CMR.15.1.111-124.2002 PMC118063

[67] Leaper D , McBain AJ , Kramer A , et al. Healthcare associated infection: novel strategies and antimicrobial implants to prevent surgical site infection. Ann R Coll Surg Engl. 2010;92(6):453‐458. 20819330 10.1308/003588410X12699663905276 PMC3182781

[68] Fay A , Nallasamy N , Bernardini F , et al. Multinational comparison of prophylactic antibiotic use for eyelid surgery. JAMA Ophthalmol. 2015;133(7):778‐784. 25905446 10.1001/jamaophthalmol.2015.0789

