# Supplementary material for: Ultra‐thin, high strength, antibiotic‐eluting sutures for prevention of ophthalmic infection
Source: Bioeng Transl Med. 2020 Dec 9;6(2):e10204. doi: 10.1002/btm2.10204 (PMC8126818; doi:10.1002/btm2.10204)
Supplement: Supplementary file 1 — Appendix S1: Supporting information [file BTM2-6-e10204-s001.pdf]

Ultra-thin, High Strength, Antibiotic-eluting Sutures for Prevention of Ophthalmic Infection

Kunal S. Parikh[1,2,3,4], Revaz Omiadze[1,2], Aditya Josyula[1,5], Richard Shi[1,4], Nicole M. Anders[6], Ping He[6], Youseph Yazdi[3,4], Peter J. McDonnell[2], Laura M. Ensign[1,2,4,5]*, Justin Hanes[1,2,4,5,6]*

Affiliations:
[1] Center for Nanomedicine, The Wilmer Eye Institute, Johns Hopkins University School of Medicine, 400 North Broadway, Baltimore, MD 21231, USA

[2] Department of Ophthalmology, The Wilmer Eye Institute, Johns Hopkins University School of Medicine, 600 North Wolfe Street, Baltimore, MD 21287, USA

[3] Center for Bioengineering Innovation & Design, Johns Hopkins University, 3400 North Charles Street, Baltimore, MD 21218, USA

[4] Department of Biomedical Engineering, Johns Hopkins University School of Medicine, 720 Rutland Avenue, Baltimore, MD 21205, USA

[5] Department of Chemical and Biomolecular Engineering, Johns Hopkins University, 3400 North Charles Street, Baltimore, MD 21218, USA

[6] Department of Oncology, Sidney Kimmel Comprehensive Cancer Center, Johns Hopkins University School of Medicine, 401 North Broadway, Baltimore, MD 21231, USA

*To whom correspondence should be addressed:
Laura Ensign (lensign@jhmi.edu) or Justin Hanes (hanes@jhmi.edu)
Center for Nanomedicine
6001 Smith Building,
400 North Broadway
Baltimore, MD 21231, USA

Supplementary Data

Table S1. In vitro breaking strength retention of nanofiber-based PCL/8% Levo after 31 and 365 days.

**Suture Type**	**Breaking Strength Retention**	
**PCL/8% Levo**	31 days	96%
**PCL/8% Levo**	365 days	75%

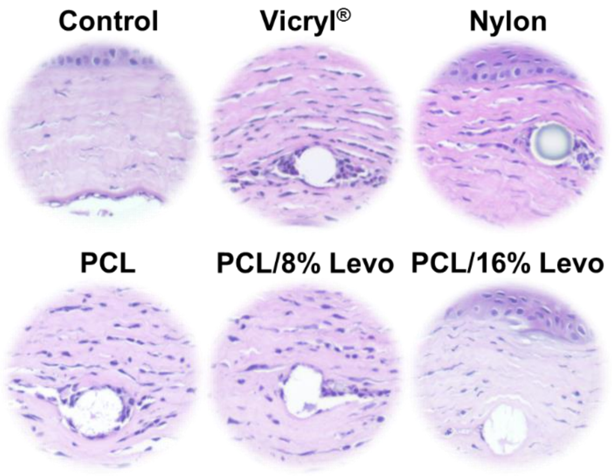

Figure S1. Histological analysis of commercially available and nanofiber-based suture biocompatibility.

Representative images of H&E-stained tissue surrounding Vicryl® and nylon sutures, and nanofiber-based PCL, PCL/8% Levo, and PCL/16% Levo sutures implanted into rat corneas for 2 days. Control indicates healthy, untreated corneal tissue.

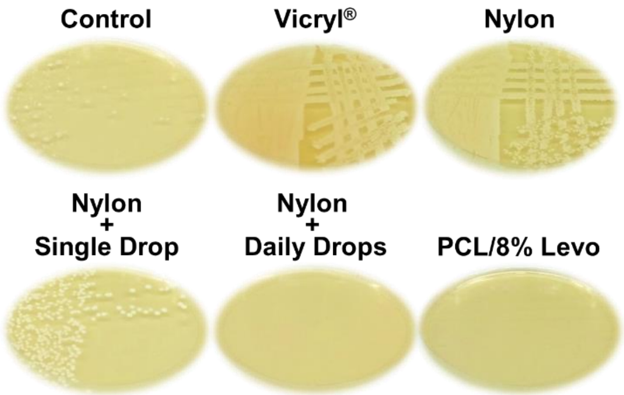

Figure S2. Evaluation of ophthalmic infection 2 days after inoculation of *S. aureus.*

Representative images of cultured bacterial swab of rat eyes 2 days after suture implantation and bacterial inoculation.

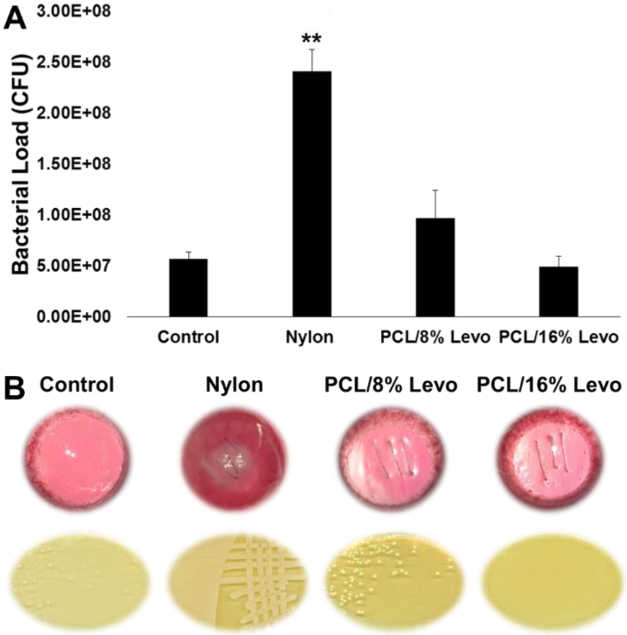

Figure S3. Evaluation of ophthalmic infection following consecutive *S. aureus* inoculations over the period of 1 week.

(A) Concentration of bacteria in inoculated corneas containing either nylon or nanofiber-based PCL/8% Levo or PCL/16% Levo sutures on day 7 after a single (nylon on day 5) or consecutive inoculations (PCL/8% Levo or PCL/16% Levo on day 0 and day 5), ** $p < 0.01$ against each other condition. **(B)** representative images of rat eyes and cultured bacterial swab of rat eyes on day 7.
